# Supplementary material for: Health Risk Assessments of Selected Trace Elements and Factors Associated with Their Levels in Human Breast Milk from Pretoria, South Africa
Source: Int J Environ Res Public Health. 2021 Sep 16;18(18):9754. doi: 10.3390/ijerph18189754 (PMC8464967; doi:10.3390/ijerph18189754)
Supplement: Supplementary file 1 [file ijerph-18-09754-s001.zip › ijerph-1347220-supplementary.pdf]

Supporting Information

Health Risk Assessments of Selected Trace Elements and Factors Associated with Their Levels in Human Breast Milk from Pretoria, South Africa

Joshua O. Olowoyo [1]*, Linda R. Macheka [1] and Phiona Moloi [1]

[1] Department of Biology, Sefako Makgatho Health Sciences University, P.O Box 139, Pretoria, South Africa and 0204
* Correspondence: woleolowoyo@yahoo.com

Table S1: Correlation of trace element levels with maternal and infant characteristics

		Maternal Age	Maternal Weight	Marital Status	Maternal Education	Employment Status	Monthly Household Income	Parity	Maternal Passive Smoking	Infant Birth Weight	Infant Sex	Birth Abnormalities	Infant Feeding Frequency
Co	Correlation Coefficient	0.047	-0.068	0.012	-0.116	0.141	-0.163	-0.172	-0.026	0.303*	-0.223	-0.127	0.006
	Sig. (1-tailed)	0.367	0.313	0.466	0.202	0.155	0.119	0.107	0.427	0.013	0.052	0.180	0.483
Cr	Correlation Coefficient	-0.078	-0.116	-0.073	-0.136	0.074	-0.056	-0.138	-0.050	0.006	-0.147	-0.016	-0.013
	Sig. (1-tailed)	0.287	0.201	0.301	0.164	0.297	0.344	0.160	0.360	0.482	0.144	0.454	0.462
As	Correlation Coefficient	-0.026	-0.163	-0.096	-0.063	0.042	-0.086	-0.040	-0.030	0.044	-0.086	-0.129	-0.011
	Sig. (1-tailed)	0.425	0.119	0.245	0.325	0.381	0.267	0.387	0.414	0.377	0.268	0.175	0.468
Pb	Correlation Coefficient	-0.042	-0.118	-0.119	-0.077	0.137	-0.062	-0.066	-0.127	0.002	-0.130	-0.060	-0.059
	Sig. (1-tailed)	0.382	0.197	0.196	0.290	0.162	0.328	0.317	0.180	0.494	0.174	0.334	0.336
Cd	Correlation Coefficient	-0.038	-0.137	0.139	-0.062	0.336**	-0.129	-0.131	-0.124	0.064	-0.190	0.130	-0.094
	Sig. (1-tailed)	0.391	0.161	0.158	0.327	0.006	0.176	0.173	0.187	0.323	0.084	0.175	0.250
Mn	Correlation Coefficient	0.076	-0.074	0.050	-0.148	0.053	-0.110	0.027	0.337**	0.172	-0.226	-0.147	-0.130
	Sig. (1-tailed)	0.293	0.298	0.359	0.143	0.353	0.214	0.422	0.006	0.106	0.050	0.144	0.175

**Correlation is significant at the 0.01 level (1-tailed). *Correlation is significant at the 0.05 level (1-tailed).

Table S2: Correlation of trace element levels with maternal diet

		Fresh Fish	Tinned Fish	Sea Food	Dairy Milk	Egg	Drinking Water Source
Co	Correlation Coefficient	0.068	0.048	0.081	0.065	-0.049	-0.115
	Sig. (1-tailed)	0.311	0.365	0.280	0.321	0.361	0.203
Cr	Correlation Coefficient	0.043	0.248*	0.384**	0.239*	0.073	0.070
	Sig. (1-tailed)	0.379	0.035	0.002	0.041	0.301	0.307
As	Correlation Coefficient	0.053	0.066	0.252*	0.080	-0.019	0.029
	Sig. (1-tailed)	0.351	0.319	0.033	0.283	0.445	0.416
Pb	Correlation Coefficient	0.058	0.005	0.290*	0.067	-0.045	0.051
	Sig. (1-tailed)	0.340	0.485	0.017	0.316	0.372	0.356
Cd	Correlation Coefficient	0.153	-0.075	0.228*	0.119	-0.123	0.089
	Sig. (1-tailed)	0.134	0.295	0.049	0.195	0.188	0.260
Mn	Correlation Coefficient	0.035	0.079	0.300*	0.047	0.013	0.074
	Sig. (1-tailed)	0.401	0.286	0.014	0.368	0.464	0.297

**Correlation is significant at the 0.01 level (1-tailed). *Correlation is significant at the 0.05 level (1-tailed).
